# Improvement of the gait pattern after selective dorsal rhizotomy derives from changes of kinematic parameters in the sagittal plane

**DOI:** 10.3389/fped.2022.1047227

**Published:** 2022-12-23

**Authors:** Wenbin Jiang, Shuyun Jiang, Yan Yu, Qijia Zhan, Min Wei, Rong Mei, Fang Chen, Yao Guo, Bo Xiao

**Affiliations:** ^1^ Department of Neurosurgery, Shanghai Children’s Hospital, School of Medicine, Shanghai Jiao Tong University, Shanghai, China; ^2^ Department of Gait Analysis, Yueyang Hospital of Integrated Traditional Chinese and Western Medicine, Shanghai University of Traditional Chinese Medicine, Shanghai, China; ^3^ Department of Urology, Shanghai Jiao Tong University Affiliated Sixth People’s Hospital, Shanghai, China; ^4^ Institute of Medical Robotics, Shanghai Jiao Tong University, Shanghai, China

**Keywords:** spastic cerebral palsy, selective dorsal rhizotomy, gait analysis, gait deviation index, statistical parametric mapping

## Abstract

**Objective:**

Selective dorsal rhizotomy (SDR) can decrease spasticity in children suffering from spastic cerebral palsy (SCP) and thus improve their moving ability when supplemented with the post-operational rehabilitation program. In this case, the study aims to investigate the gait changes in children with mild SCP after SDR in short-term follow-up.

**Methods:**

The information of ambulatory SCP cases who underwent SDR in our center was retrospectively reviewed, and comparisons of changes in spasticity, motor function and data of gait analysis before and after SDR were analyzed.

**Results:**

In total, 32 cases were included in this study, with a mean age of 5.9 ± 2.1 years old. Noticeable decrease was found in the median value of the pre-operational MAS score after SDR at last follow-up in both sides of adductors, gastrocnemius, soleus, and left hamstrings. The Gross Motor Function Measure-66 score increased from 70.6 ± 9.2 to 73.4 ± 8.2, and the gait deviation index increased after SDR compared with the pre-operational data (right side: 65.8 ± 8.8 vs. 60.1 ± 10.7; left side: 63.5 ± 10.1 vs. 57.0 ± 9.9). Noticeable changes were found that the maximum angle of affected ankles in the sagittal plane (the dorsal-flexion angle) increased from 2.5° to 8.2°, the angles at initial contact (1% gait cycle) of affected knees in the sagittal plane decreased from 34.0° to 27.8°, and the angles at the end of swing phase (100% gait cycle) of affected knees in the sagittal plane decreased from 35.8° to 28.3°.

**Conclusion:**

In short-term follow-up, SDR can lower spasticity in children with SCP. Post-operational gait analysis showed improvements in gross motor function and gait, which derived from the changes in the sagittal plane (ankle and knee). A longer follow-up duration is thus needed to clarify the long-term outcome.

## Introduction

Selective dorsal rhizotomy (SDR) is a neurosurgical intervention primarily applied to patients suffering from spastic cerebral palsy (SCP), which is usually performed at the lumbosacral level, and can reduce spasticity in the lower extremities (1). During the operation, certain dorsal rootlets were selected and partially transected to decrease sensory inputs (2, 3). The results showed that SDR could be applied in cases with severe generalized spasticity, which in detail, patients classified as Gross Motor Function Classification System (GMFCS) Level IV and V). Except for that, SDR worked well in children with focal spasticity as well, since the therapeutic effectiveness of spastic hemiplegic cases after SDR has been previously reported, implying its potential to treat mild SCP (4, 5). As a result, SDR might also be a good choice for ambulatory SCP cases, though rehabilitation, soft-tissue surgeries, or orthotics were previously more preferred for improving the gait patterns and motor function of these patients (6–8).

The aim of treating children with SCP is to help these children live independently by improving their moving ability, making it crucial not only to relieve the spasticity, but also to promote their walking ability (9). The current study aims to investigate the short-term gait parameter changes assessed by gait analysis after SDR in mild SCP cases.

## Methods and materials

A cohort review was hereby conducted in children diagnosed with SCP who had accepted SDR in Shanghai Children's Hospital from Jul. 2017 to Aug. 2019. The diagnosis of SCP was made by our multidisciplinary team composed of physiotherapists, specialists in gait analysis, and neurosurgeons. Clinical data including demographics and relevant medical records were taken from the Database of Pediatric Cerebral Palsy of the Department.

### Inclusion criteria

Ambulatory SCP (mainly cases classified as GMFCS level I and level II, with a few classified as level III); No structural orthopedic deformities or fixed tendon contractures; Aged between 3 and 14 years old at SDR procedure; Good cognitive ability of children, and good support from parents and rehabilitation settings; Having experienced pre-op gait analysis evaluation and at least once post-op gait analysis assessment; No extra surgical interventions before SDR other than rehabilitation program.

### Assessment of spasticity

Muscle tone of bilateral lower extremities in all patients was assessed by one physiotherapist before SDR and at the follow-up using the modified Ashworth Scale (MAS) score (10), which is generally used to determine the MAS grade (11), with the explicit definition explained in Supplementary Table S1. Hereby the assessed muscles included bilateral hip adductors, hamstrings, gastrocnemius, and soleus. Muscles evaluated as MAS score 3 or higher (≥grade 2 of Modified Ashworth grading scale) before the SDR procedure were referred to as the target muscles.

### Evaluation of motor function

The motor function of all patients was examined by the very physiotherapist who conducted the muscle tone assessment. The GMFCS and gross motor function measure-66 (GMFM-66) were utilized to assess the motion ability of the participants (12–14). GMFCS is a five-grade classification system for determining the motor function of patients presenting from level I to level V (Supplementary Table S1), while GMFM-66 is an observational clinical tool for the evaluation of motor function changes in SCP patients. The GMFM-66 scoring system is a four-point-scale system comprising 66 items grouped into five dimensions of gross motor function. A 5-year-old child without motor disabilities exhibits the maximum score (a score of 100). Besides, the GMFM-66 score is highly correlated with the GMFCS grade, but the score is more accurate as a tool for motor function evaluation compared with the use of the classification system.

### SDR procedure and post-operative rehabilitation program

All cases underwent SDR through a single-level approach (L2–L3) similar to the surgical technique reported by Park and Johnston (15), and the rhizotomy protocol was described in detail in our previous articles (16). The post-operative rehabilitation program, which was mentioned in our previous article, were applied to these children after SDR. In detail, strengthening program starts 3 days after the operation, and the balancing program starts 7 days after SDR. Ambulating program starts 3, 6 and 12 months after SDR in children classified as GMFCS I, II and III, respectively. Gaiting program starts 6, 9 and 18 months after SDR in children classified as GMFCS I, II and III, respectively (Supplementary Figure S1).

### Gait analysis and gait deviation index

All cases included in this study received a comprehensive gait analysis evaluation, and all the gait analysis data were collected using a twelve-camera Motion Analysis System (Cortex 8, Motion Analysis Corporation, Santa Rosa, United States). The 3D coordinates of markers were used as inputs to a commercial software program (Visual3D, C-Motion, MD), while the Visual 3D program was used for defining the joint centers and segment coordinate systems from the 3D marker trajectories and the subsequent rigid body kinematic calculations. Changes in kinematic data of the ankle, knee, and hip were correspondingly compared to investigate different outcomes of SDR in these angles, including the maximum and minimum ankle angle in the sagittal plane, and the ankle angle at the end of swing phase in the sagittal plane, the average foot progression angle, the maximum knee flexion angle, the knee flexion angle at initial contact and end of swing phase and the hip average adduction angle, the maximum hip flexion angle, as well as the hip flexion angle at initial contact and end of swing phase.

The overall kinematic in each limb was assessed using gait deviation index, which was first described by Schwartz and Rozumalski (17). It is defined as a scaled distance between 15 gait feature scores for a subject and the average of the identical 15 gait feature scores for a control group of typically developing children. The full gait deviation index score is 100 (and a score over 100 indicates a subject whose gait is as close to the typically developed individual, which indicates the absence of gait pathology). Every 10 points that the gait deviation index falls below 100 corresponds one standard deviation away from the typically developed average.

### Statistical analysis

The kinematic curves were statistically compared using the open-source 1-dimensional statistical parametric package “SPM1D” proposed by Pataky (18). All Statistical Parametric Mapping (SPM) analyses were conducted in MATLAB (Version 2021a, MathWorks Inc., Natick, MA, United States) using the software package downloaded online (www.spm1d.org). Two main types of analyses were hereby used, i.e., the SPM Hotelling's T2 tests and the SPM *t*-tests. Joint vector fields were constructed by assembling multi-component time series of all subjects, including the kinematic data of three lower joints (hip, knee, and ankle) in the sagittal, transverse and coronal plane. The post-operational kinematic curves were compared to the pre-operational ones using the vector-field (multi-variate) equivalent of the paired *t*-test, a paired Hotelling's T2 test, and the exact process was used for *post hoc* comparisons (SPM *t*-tests), considering each kinematic data in the ankle, knee, and hip before and after the surgery. The results were output by the MATLAB program.

MATLAB and commercial statistical software (SPSS version 25.0, IBM) were used for statistical analyses. As mentioned above, the SPM Hotelling's T2 and SPM *t*-tests in the “SPM1D” package were carried out to compare the differences between pre-op and post-op kinematic curves in the sagittal plane of the ankle, knee, and hip, and the grey bars in the output pictures indicate regions with significant statistical differences. Standard distribution data are presented as mean ± SD, and non-normal distribution data are presented as median (Q1, Q3). Changes of muscle tension were compared using Wilcoxon matched-pair signed-rank test, and pre-post comparisons of GMFM-66 score, kinematic and temporal-spatial parameters after SDR were assessed using the matched-pair *T*-test. Besides, the linear association between gait deviation index change and other variables were assessed using Spearman's correlation. A statistical significance level of *p* < 0.05 was set up for all tests.

## Results

A total of 32 cases (26 boys, 6 girls) were included in this study (Table 1), aged from 3 to 12 at SDR, with a mean of 5.9 ± 2.1 years old. The duration of post-operational follow-up was between 379 ± 187 days. Among these cases, 13 (40.6%) were classified as GMFCS level I; 14 (43.8%), level II; and 5 (15.6%), level III, respectively. Regarding the type of SCP, 8 patients were monoplegia or hemiplegia, while other 24 were diplegia. The 8 limbs of these 32 patients without abnormal muscle tension were marked as the intact sides, while considering the elevated muscle tension, other 56 limbs were taken as the affected sides. In general, the median number of nerve roots (rootlets) transected during SDR was 7, with a range from 2 to 19. An average of 9 roots (rootlets) were cut in 24 diplegic children, ranging from 5 to 19, covering both sides. While in 5 right hemi-/monoplegic and 3 left hemi-/monoplegic children, a mean of 3 roots (rootlets) were cut only in affected sides. No surgical-related complications, except hypersensitivity in 5 cases, were observed during the last follow-up within two weeks after the operation.

**Table 1:** Demographic and clinical data of cases included in this study.

Characteristics	
**Gender (*n*, %)**	
* Boy*	26 (81.2%)
* Girl*	6 (18.8%)
**Age at surgery (years old)**	5.9 ± 2.1
**SCP type (*n*, %)**	
* Monoplegia*	4 (12.5%)
* Hemiplegia*	4 (12.5%)
* Diplegia*	24 (75.0%)
**Pre-op GMFCS level (*n*, %)**	
* I*	13 (40.6%)
* II*	14 (43.8%)
* III*	5 (15.6%)
**Pre-op GMFM-66 score (mean **±** SD)**	70.6 ± 9.2
**Post-op GMFM-66 score (mean ± SD)**	73.4 **±** 8.2
**Follow-up (days, mean ± SD)**	379 ± 187
**Number of target muscles (*n*)**	
* Adductors*	34
* Hamstrings*	29
* Gastrocnemius*	57
* Soleus*	52
**Number of nerve roots (rootlets) cut during SDR (median, range)**	
* Left*	3.5 (0–11)
* Right*	4 (0–9)

SCP, spastic cerebral palsy; GMFCS, gross motor function classification system; GMFM-66, gross motor function measurement-66; SDR: selective dorsal rhizotomy.

**Figure 1:**
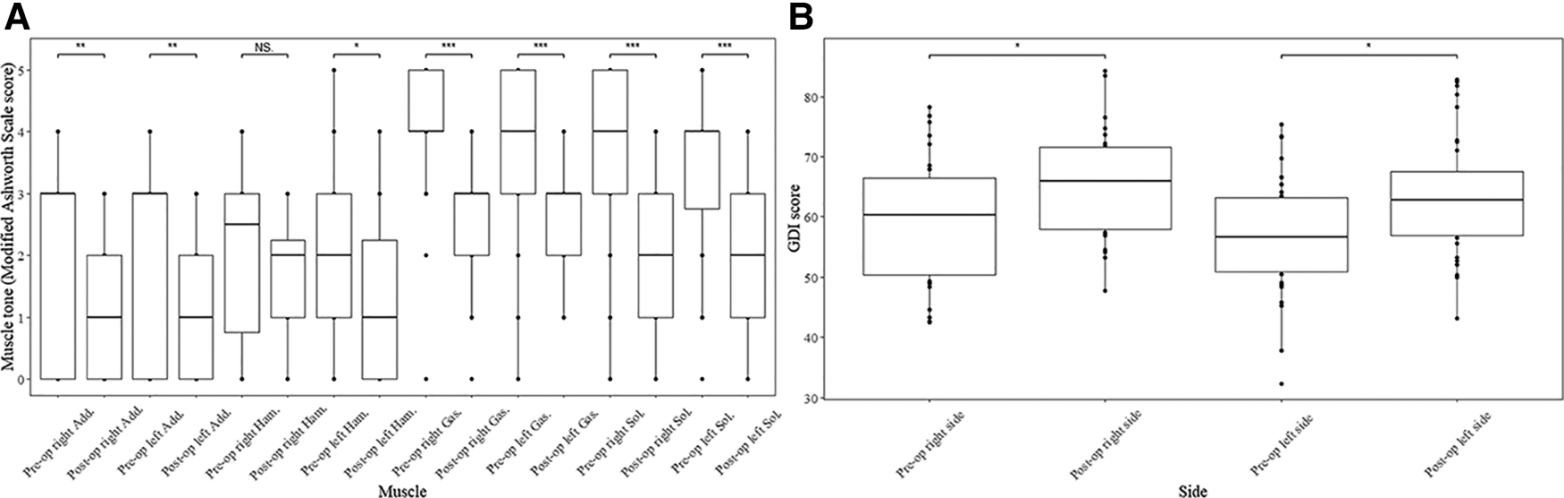
Muscle tone of muscles and gait deviation index (GDI) score of both limbs before and after SDR. Muscle tone change of muscles at both sides of lower limbs after selective dorsal rhizotomy assessed by modified Ashworth Scale score (**A**) and GDI score change of both sides after the surgery (**B**). Add., adductors; Ham., hamstring; Gas., gastrocnemius; Sol., soleus; GDI, gait deviation index; SDR, selective dorsal rhizotomy. *p < 0.05, **p < 0.01, ***p < 0.001.

Noticeable decrease was observed in the median value of the post-operational MAS score after SDR at last follow-up (Figure 1) in both sides of adductors (right side: score 3 vs. score 1, *p* < 0.01; left side: score 3 vs. score 1, *p* < 0.01), gastrocnemius (both side: score 4 vs. score 3, *p* < 0.001), soleus (both side: score 4 vs. score 2, *p* < 0.001), and left hamstrings (score 2 vs. score 1, *p* < 0.05). The median muscle tone of right hamstrings was unchanged (score 2.5 vs. score 2). After the surgery, the GMFM-66 score increased from 70.6 ± 9.2 to 73.4 ± 8.2 (Table 1, *p* < 0.01) at the last follow up, and the gait deviation index increased after SDR compared with the pre-operational data (Figure 1, right side: 65.8 ± 8.8 vs. 60.1 ± 10.7, *p* < 0.05; left side: 63.5 ± 10.1 vs. 57.0 ± 9.9, *p* < 0.05).

The correlation between the change of the gait deviation index scores with other separate variables in both sides is listed in Table 2. The change of gait deviation index in the right side was found highly correlated with the follow-up duration after SDR (*r* = 0.83, *p* < 0.0001), and this correlation could also be observed in the left side (*r* = 0.60, *p* < 0.001). Nonetheless, the change of gait deviation index in both lower limbs had no correlation with the age at surgery and pre-operational GMFM-66 score.

**Table 2:** Correlation between gait deviation index (GDI) change and different variables.

	Change of GDI in right sides		Change of GDI in left sides		Change of GDI in affected sides		Change of GDI in intact sides	
	*r*	*p* value	*r*	*p* value	*r*	*p* value	*r*	*p* value
Age at surgery	−0.11	0.53	−0.06	0.73	−0.08	0.58	−0.36	0.38
Pre-op GMFM-66 score	−0.19	0.36	0.17	0.37	0.02	0.89	−0.26	0.54
Follow-up duration	0.83	**<0**.**0001**	0.60	**<0**.**001**	0.68	**<0**.**0001**	0.69	0.07

Bold values indicate statistical significance.

**Figure 2:**
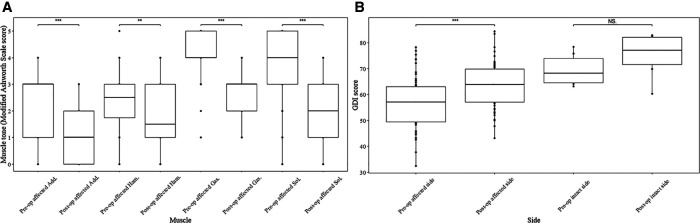
Muscle tone of muscles in affected limbs and GDI of both affected and intact limbs before and after SDR. Muscle tone change of muscles at affected sides after selective dorsal rhizotomy assessed by modified Ashworth Scale score (**A**) and the gait deviation index score change of both affected and intact limbs after the surgery (**B**). Add., adductors; Ham., hamstring; Gas., gastrocnemius; Sol., soleus; GDI, gait deviation index; SDR, selective dorsal rhizotomy; NS, no statistical significance. *p < 0.05, **p < 0.01, ***p < 0.001.

Given that 8 cases were hemiplegic and monoplegic, the changes of the MAS score in 56 affected limbs (Figure 2) and 8 intact limbs (Supplementary Figure S2) were further compared. The median of the pre-operational MAS score after SDR decreased significantly in the muscles of all affected sides (Figure 2), and the specific data were: adductors (score 3 vs. score 1, *p* < 0.001), hamstrings (score 2.5 vs. score 1.5, *p* < 0.05), gastrocnemius (score 4 vs. score 3, *p* < 0.001), and soleus (score 4 vs. score 2, *p* < 0.001). On the contrary, the muscle tone remained unchanged in the intact limbs (Supplementary Figure S2). The gait deviation index of the affected sides increased after SDR compared with the pre-operational data (Figure 2, 63.1 ± 8.7 vs. 57.0 ± 9.9, *p* < 0.001), and the gait deviation index of the intact sides also increased after SDR though no statistical difference was observed (75.4 ± 8.0 vs. 69.4 ± 5.8).

The change of the gait deviation index scores in the affected sides was highly correlated with the follow-up duration after SDR (Table 2, *r* = 0.68, *p* < 0.0001). Though the *r* value between follow-up duration and gait deviation index change was high in intact sides, statistical significance does not exist (*r* = 0.69, *p* = 0.07).

**Figure 3:**
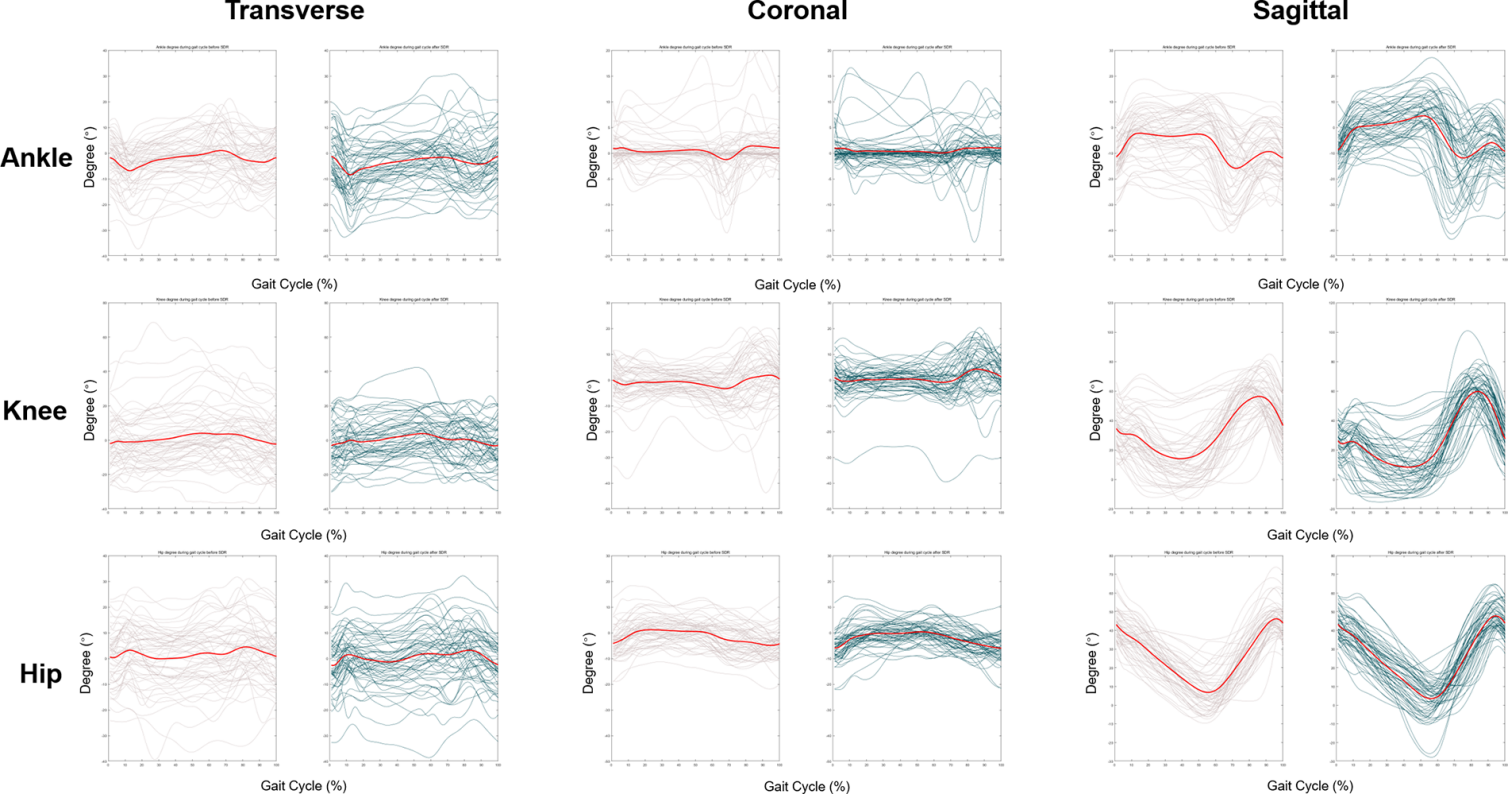
Kinematic curves of ankle, knee and hip in affected lower extremities at transverse, coronal and sagittal plane. Transverse plane: internal rotation and external rotation. Before the surgery, the median degree of ankle, knee, hip ranged from −2.1° to 3.3°, −6.2° to 5.8°, −8.3° to 3.3° in transverse plane, respectively. After SDR, the median degree of ankle, knee, hip ranged from −11.8° to 3.1°, −8.9° to 8.7°, −5.7° to 7.0° in transverse plane, respectively. Positive value means internal rotation; negative value means external rotation. Coronal plane: abduction and adduction. Before the surgery, the median degree of ankle, knee, hip ranged from −7.4° to 3.9°, −8.5° to 5.7°, −3.3° to 6.6° in coronal plane, respectively. After SDR, the median degree of ankle, knee, hip ranged from −1.9° to 1.3°, −3.6° to 7.3°, −8.0° to 2.3° in coronal plane, respectively. Positive value means abduction; negative value means adduction. Sagittal plane: flexion and extension or dorsiflexion and plantarflexion (ankle). Before the surgery, the median degree of ankle, knee, hip ranged from −18.6° to 2.5°, 7.9° to 61.9°, 4.0° to 45.7° in sagittal plane, respectively. After SDR, the median degree of ankle, knee, hip ranged from −14.6° to 8.2°, 2.8° to 62.2°, 4.5° to 47.1° in sagittal plane, respectively. Positive value means dorsiflexion; negative value means plantarflexion. Grey lines: pre-operational status, green lines: post-operational status, red lines: average kinematic curve.

**Figure 4:**
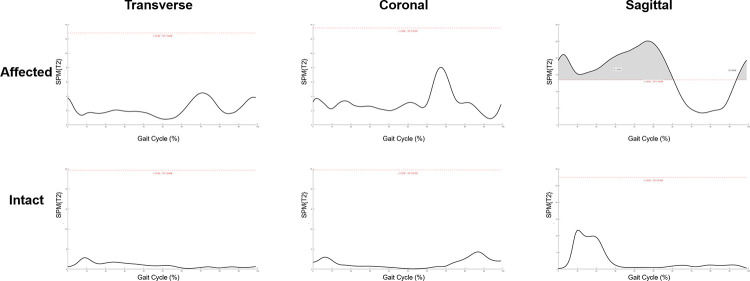
Statistical parametric mapping Hotellings2 tests of pre-operational and post-operational kinematic curves in both affected and intact sides at transverse, coronal and sagittal plane. Grey region above the red dash line: statistical significance region.

Change of temporal-spatial parameters were listed in Supplementary Table S2. No other significant change was found after SDR except for the decrease in cadence from 119.9 ± 26.5 steps/min to 108.1 ± 24.4 steps/min (*p* < 0.05). The kinematic curves of the affected sides in transverse, coronal and sagittal planes before and after SDR are demonstrated in Figure 3, while those of the intact sides are listed in Supplementary Figure S3. To figure out the difference between pre-operational and post-operational kinematic curves, the SPM hotellings2 tests were performed and the results were shown in Figure 4. The upper panel of Figure 4 showed that statistical difference existed in the sagittal plane from 1% to 60.5% (*p* < 0.001) gait cycle and 93.5%–100% gait cycle (*p* < 0.05), while no differences were observed when comparing other planes of affected sides and all three planes of intact sides.

**Figure 5:**
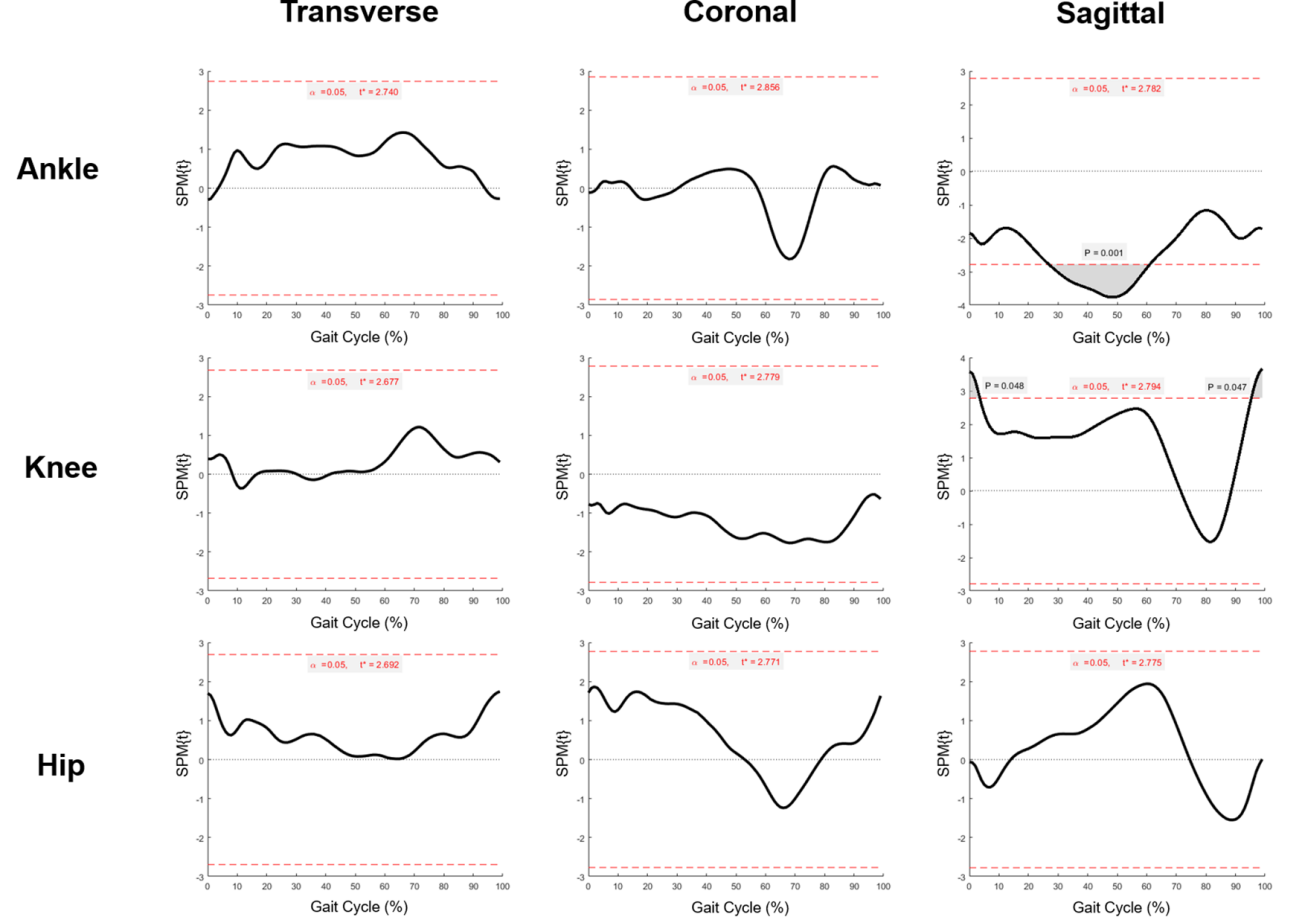
Statistical parametric mapping *t* tests of pre-operational and post-operational kinematic curves (ankle, knee and hip) in affected sides at transverse, coronal and sagittal plane. Grey region above/below the red dash line: statistical significance region.

To exactly locate the changes of kinematic curves, we utilized the *post hoc t*-tests in SPM package. The difference of ankle existed in the 27%–61% gait cycle in the sagittal plane (*p* = 0.001), and the difference of knee existed in the 1%–3.5% (*p* < 0.05), and 95.5%–100% (*p* < 0.05) gait cycle in the sagittal plane (Figure 5). Comparisons of the extracted scalars also confirmed the changes in three lower joints (Table 3). Noticeable changes were found in median value that maximum angle of affected ankles in the sagittal plane (the dorsal-flexion angle) increased from 2.5° to 8.2° (*p* < 0.001), angles at initial contact (1% gait cycle) of affected knees in the sagittal plane decreased from 34.0° to 27.8° (*p* < 0.01), and angles at the end of swing phase (100% gait cycle) of the affected knees decreased from 35.8° to 28.3° (*p* < 0.01). No significant changes in the kinematics of the hip joints were revealed after SDR in these cases, and the kinematic data in the intact sides showed no statistical difference as well (Supplementary Figure S4).

**Table 3:** Changes of extracted kinematic parameters in patients included in this study.

Characteristics	Affected sides			Intact sides		
Kinematics	Pre-op	Post-op	*p* value	Pre-op	Post-op	*p* value
Ankle						
*Maximum flexion angle*	2.5 (−3.7, 7.7)	8.2 (2.6, 12.8)	**<0.001**	7.5 (5.0, 15.4)	7.3 (3.8, 9.8)	0.57
*Minimum flexion angle*	−18.6 (−29.7, −10.5)	−14.6 (−26.1, −8.0)	0.18	−12.7 (−27.6, −9.8)	−16.7 (−24.8, −14.7)	0.44
*Angle at the end of swing angle in sagittal plane* ^a^	−13.3 (−17.3, −5.9)	−8.9 (−13.5, −5.0)	0.06	−6.0 (−10.3, −2.8)	−8.6 (−12.1, −4.6)	0.57
*Average foot progression angle* ^b^	−1.6 (−6.3, 2.4)	−4.0 (−8.9, 1.8)	0.17	−2.5 (−6.9, 4.5)	−6.6 (−8.1, −0.5)	0.38
Knee						
*Flexion angle at initial contact*	34.0 (23.9, 43.6)	27.8 (17.4, 35.0)	**<0.01**	13.7 (2.8, 20.3)	10.4 (9.2, 17.6)	0.88
*Flexion angle at end of swing*	35.8 (27.1, 45.4)	28.3 (18.1, 35.9)	**<0.01**	16.7 (5.0, 21.3)	11.3 (9.5, 19.0)	0.96
*Maximum flexion angle*	61.9 (52.7, 67.5)	62.2 (58.0, 70.1)	0.32	64.1 (60.2, 66.9)	65.1 (58.4, 69.8)	0.88
Hip						
*Flexion angle at initial contact*	42.1 (37.0, 47.7)	42.6 (37.9, 48.0)	0.74	38.3 (33.8, 43.7)	38.9 (34.2, 45.8)	0.80
*Flexion angle at end of swing*	43.2 (37.6, 48.7)	42.6 (37.9, 48.0)	0.85	38.7 (34.1, 45.0)	38.6 (34.0, 46.2)	0.96
*Maximum flexion angle*	45.7 (41.7, 50.6)	47.1 (42.8, 47.1)	0.21	43.0 (35.0, 49.0)	40.0 (36.7, 51.5)	0.88
*Average abduction angle*	2.8 (−5.8, 8.7)	0.5 (−3.9, 7.0)	0.54	−7.8 (−9.1, −6.9)	−1.9 (−6.0, 0.3)	0.19

Bold values indicate statistical significance.

^a^ Positive value means dorsiflexion; negative value means plantarflexion.

^b^ Positive value means internal rotation; negative value means external rotation.

## Discussion

Gait dysfunction is an essential factor that restricts the independence and quality of life in the SCP population (9). Much emphasis should be placed on improving the walking ability of patients with SCP for the improvement of their life quality. The improved waking ability has a positive impact on the achievement of their daily activities and the motivation of their social engagement, which matters considerably for these patients (19). Additionally, spasticity is the main factor affecting the motor function of children with SCP, which could be relieved by SDR (20). Patients with SCP could improve their motor function after the reduction of spasticity if they keep doing post-operational rehabilitation (4, 5). To figure out the change of the walking ability in these patients, the pre-operational and post-operational gait analysis of ambulatory cases who accepted SDR in the center was reviewed, and these patients were mainly classified as GMFCS level I and level I, including a few classified as GMFCS level III with good moving ability, who were also ambulatory. After SDR, the spasticity of muscles in both lower extremities decreased significantly in patients diagnosed as diplegic SCP. Interestingly, the spasticity of muscles in the affected sides was found to reduce significantly in hemiplegic and monoplegic children. In unaffected sides, the muscle tension showed no obvious abnormality when evaluated before the surgery (Supplementary Figure S2), and the muscle tone remained almost unchanged after SDR. This phenomenon confirms the capability of SDR reducing spasticity as expected, indicating the applicability of SDR as an option for mild SCP cases.

Moreover, all patients included in this study kept receiving regular rehabilitation after the operation till the last follow-up. Most children gain improvement in motor function at follow-up revealed by elevation both in GMFM-66 score and gait deviation index (21–24). After the operation, 28/32 children had improvement in GMFM-66 score. In accordance, 28/32 children had gait deviation index improvement in the left sides, 28/32 children had gait deviation index improvement in right sides, which in detail, 51 affected sides and 5 intact sides had improved gait deviation index score after the surgery. The positive correlation between gait deviation index improvement and follow-up duration (also the post-op rehabilitation duration) indicates that the longer the post-operative follow-up is, the higher the gait deviation index score will be, which was consistent to the research previously reported (25). Children without gait deviation index increase after SDR have a much shorter follow-up duration than the others (less than half a year), and the gait deviation index will further progress as the increase of rehabilitation duration after the surgery.

Gait deviation index is a score derived mainly from the kinematic data, which depicts the overall status of walking ability. The kinematic curves were analyzed using the SPM1D to better locate the gait improvement. The results demonstrated that significant changes came from the change of kinematic curves in the sagittal plane, i.e., the knee and the ankle. The maximum angle of the ankle in the sagittal plane increased 5.7 degree at follow-up, suggesting that the ankle could be more dorsiflexed than that in the pre-operational state, which might be attributed to the decrease of spasticity in gastrocnemius and soleus, coinciding with previous literature (26, 27). Meanwhile, the knee was found to be less flexed than that before the operation at the beginning and end of gait cycle in the sagittal plane. The improvement in the knee flexion angle mainly resulted from the decrease in the muscle tension of the hamstrings and gastrocnemius muscles. The data also showed that the difference between the affected limbs and the intact limbs decreased after SDR compared with the dorsiflexion ankle angle.

Except for the significant change in the ankle and knee in the sagittal plane, it was also demonstrated that the foot turned to be more externally rotated after SDR though no statistical significance was observed in the affected limbs. This might be restricted by the limited number of cases. There are many factors influencing the activity of the cross-sectional ankle joint, and the muscle tension of the adductor and gastrocnemius will affect the internal and external rotation of the ankle. After the operation, the specific data suggested that the angle of the internal rotation of the tibia was changed from mild internal rotation to mild external rotation, which might be possibly attributed to the decrease in the muscle tone of the adductor and gastrocnemius muscles. Similar results could be found in research concerning orthopedic surgeries (28), while the improvement of the internal rotation of the tibia after SDR was quite satisfying the specific reasons should be further explored.

Though several limitations still exist in the current study, including its limited sample size and short follow-up time, this is the first study objectively evaluating outcomes of SDR treating mild spastic SCP in our center. Studies with larger samples and longer follow-up are the future research goals. There might be the plateau time or peak off period in the long-run after SDR, but it could not be told from the current data. Longer follow-up is needed to figure out the long-term outcome. SDR is expected to provide an additional option to those with mild spastic SCP and thereby improve their pathological gait patterns, which is of great value for both the patients and their families.

## Conclusion

The conclusion can be drawn out that SDR lowers spasticity in children with SCP in short-term follow-up, that the walking ability of children with SCP can be improved after SDR with the supplementation of post-op rehabilitation, and that the improvement of gait comes from changes of the ankle and knee in the sagittal plane. To this end, a longer follow-up duration is needed to clarify the long-term outcome.

## Data Availability

The raw data supporting the conclusions of this article will be made available by the authors, without undue reservation.
