# Supplementary material for: Improvement of the gait pattern after selective dorsal rhizotomy derives from changes of kinematic parameters in the sagittal plane
Source: Front Pediatr. 2022 Dec 23;10:1047227. doi: 10.3389/fped.2022.1047227 (PMC9822718; doi:10.3389/fped.2022.1047227)
Supplement: Supplementary file 1 [file Datasheet1.docx]

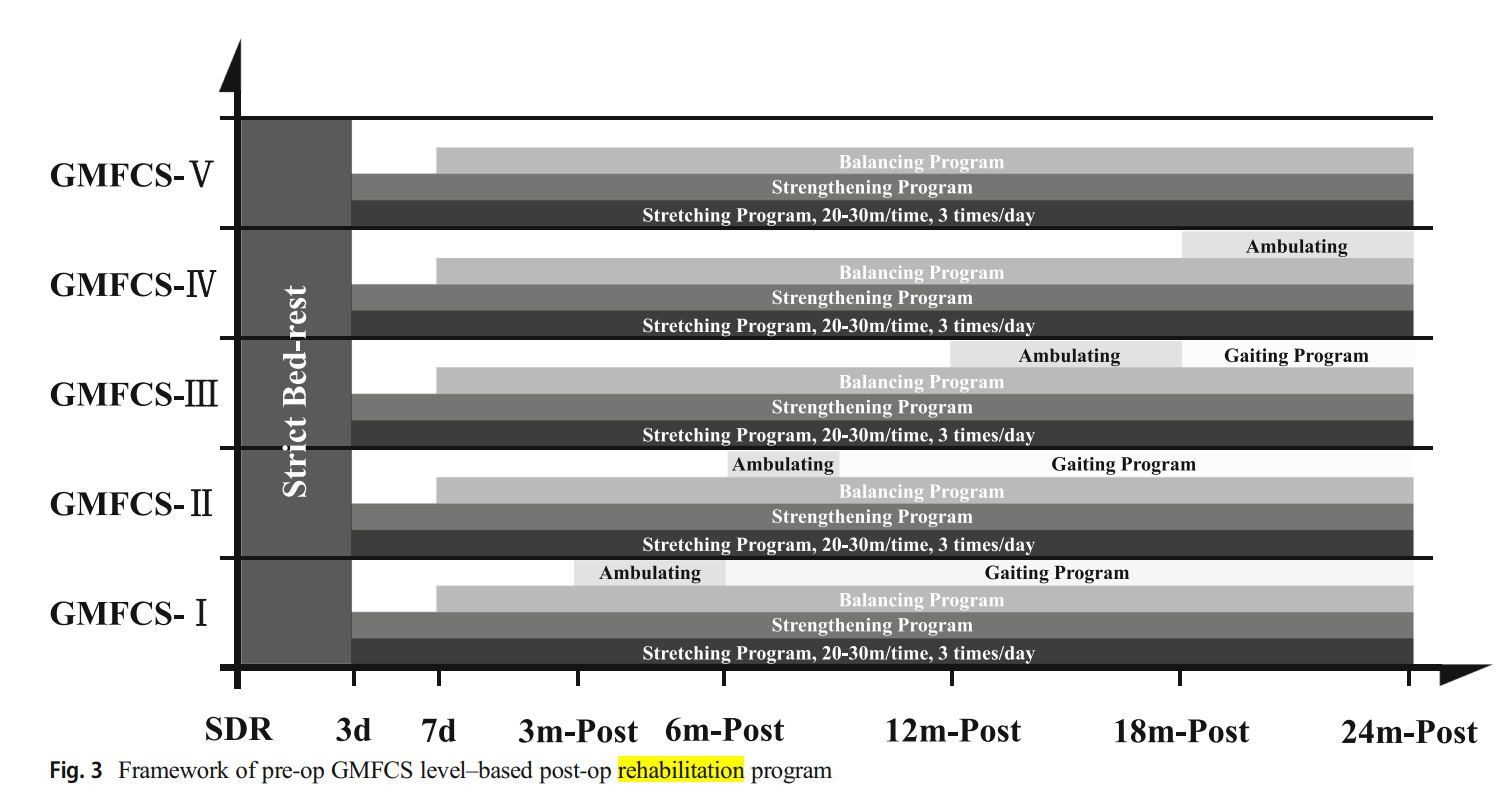


**Supplementary Figure 1.** Framework of the post-SDR rehabilitation program based on the pre-operational GMFCS level.


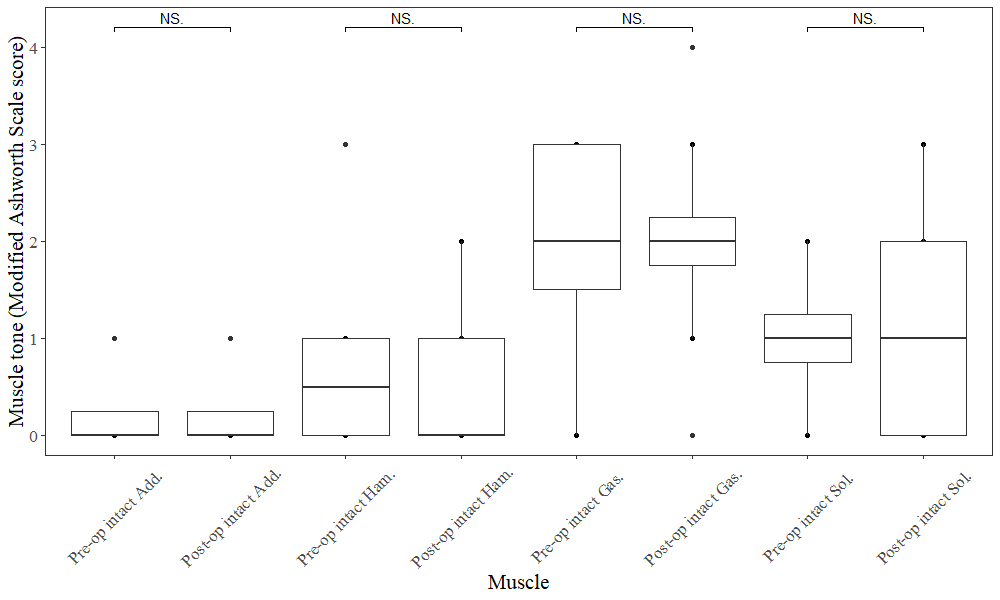


**Supplementary Figure 2.** Muscle tension change of muscles at intact limbs after selective dorsal rhizotomy assessed by Modified Ashworth Scale score.

Abbreviations:

Add.: adductors, Ham.: hamstring, Gas.: gastrocnemius, Sol.: soleus


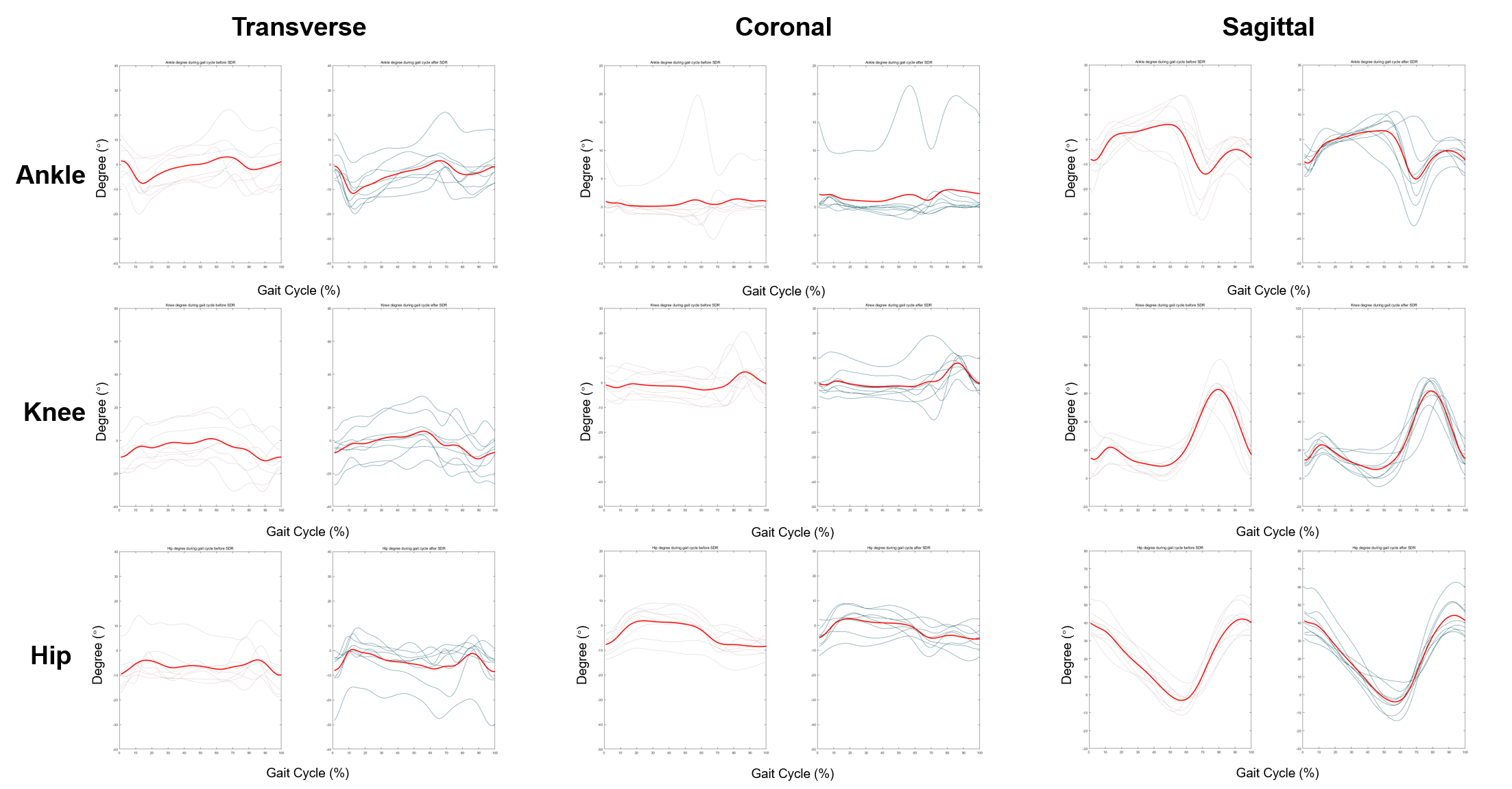


**Supplementary figure 3.** Kinematic curves of ankle, knee and hip in intact lower extremities at transverse, coronal and sagittal plane.

Grey lines: pre-operational status, green lines: post-operational status, red lines: average kinematic curve.


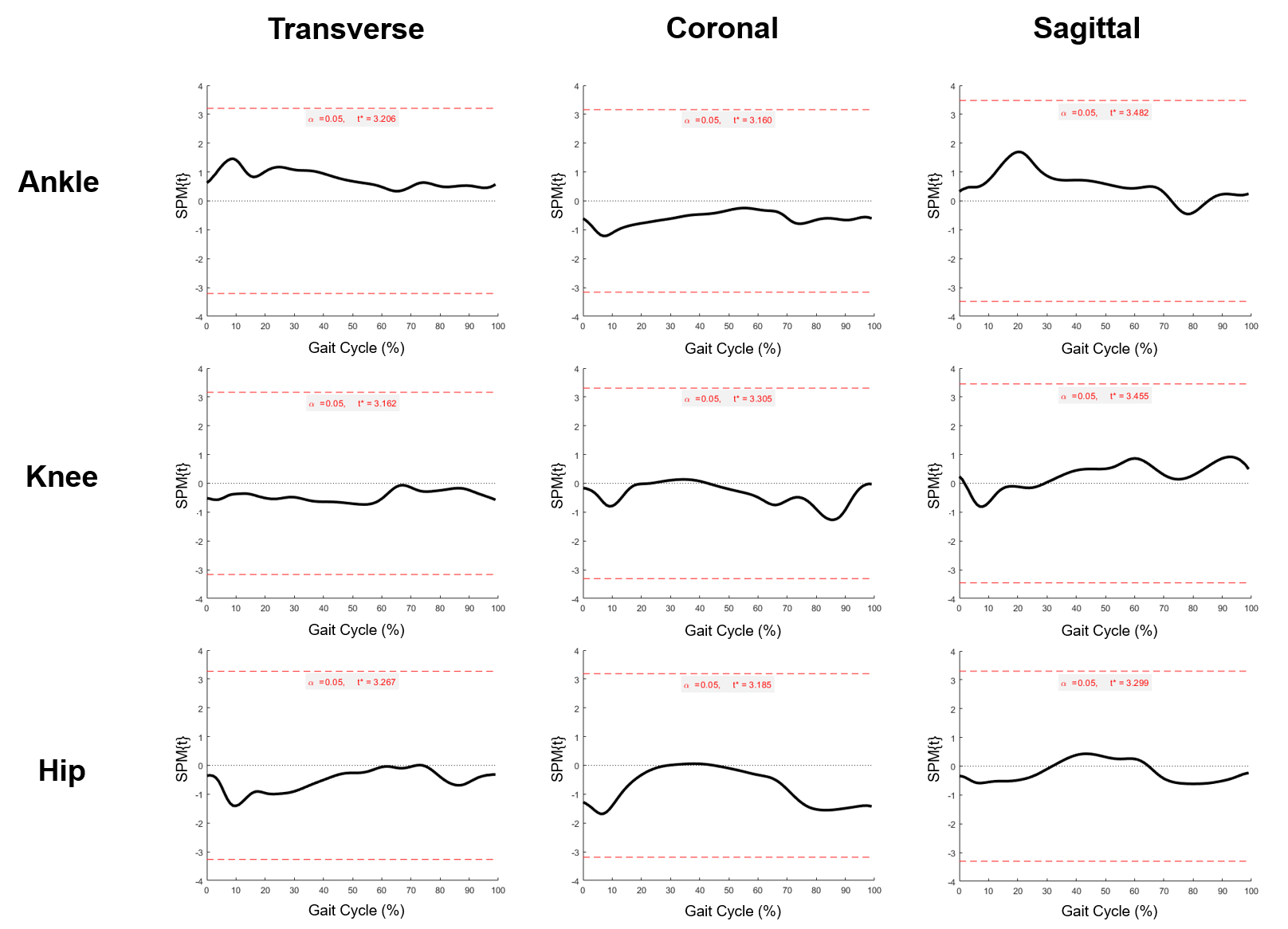


**Supplementary Figure 4.** Statistical parametric mapping t tests of pre-operational and post-operational kinematic curves (ankle, knee and hip) in intact sides at transverse, coronal and sagittal plane.

**Supplementary Table 1**. Definition of modified Ashworth Scale, modified Ashworth Scale score and gross motor function classification system.

| Modified Ashworth Scale | Modified Ashworth Scale score | Definition | Gross Motor Function Classification System | Definition |
| --- | --- | --- | --- | --- |
| 0 | 0 | No increase in muscle tone | Ⅰ | Walk without limitations |
| 1 | 1 | Slight increase in muscle tone, presented as a catch and release or by minimum resistance at the end of the range of motion when the affected part is moved in flexion or extension | Ⅱ | Walk with limitations |
| 1+ | 2 | Slight increase in muscle tone, exhibited as a catch, followed by minimal resistance throughout the remainder of the range of movement | Ⅲ | Walk with handheld equipment |
| 2 | 3 | Moderate increase in muscle tone | Ⅳ | Self-mobile with limitations |
| 3 | 4 | Significant increase in muscle tone | Ⅴ | Dependent on humans and equipment |
| 4 | 5 | Affected part in rigid flexion or extension | / | / |

**Supplementary Table 2.** Change of temporal-spatial parameters and GDI in all 32 patients in this study.

| **Temporal-spatial Characteristics** | | **Pre-op (n = 32)** | | **Post-op (n = 32)** | | ***p* value** |
| --- | --- | --- | --- | --- | --- | --- |
| *Step width, cm* | | 14.9 ± 2.9 | | 14.9 ± 3.1 | | 0.942 |
| **Temporal-spatial Characteristics** | **Affected sides (n = 56)** | | | **Intact sides (n = 8)** | | |
|  | **Pre-op** | **Post-op** | ***p* value** | **Pre-op** | **Post-op** | ***p* value** |
| *Step length, cm* | 29.9 ± 9.4 | 30.9 ± 8.2 | 0.828 | 36.0 ± 10.5 | 38.0 ± 10.8 | 0.645 |
| *Forward velocity, cm/s* | 61.9 ± 24.6 | 57.8 ± 21.8 | 0.223 | 80.7 ± 20.9 | 77.1 ± 26.9 | 0.959 |
| *Cadence, steps/min* | 119.9 ± 26.5 | 108.1 ± 24.4 | **0.014** | 137.4 ± 12.3 | 121.7 ± 25.0 | 0.382 |
| *Total support time, %* | 67.8 ± 6.9 | 68.3 ± 6.89 | 0.381 | 65.5 ± 3.9 | 65.8 ± 4.6 | 0.878 |
| *Single support time, %* | 31.5 ± 6.7 | 30.7 ± 6.24 | 0.305 | 36.8 ± 2.9 | 37.3 ± 4.6 | 0.505 |
| *Swing phase time, %* | 32.2 ± 6.9 | 31.7 ± 6.9 | 0.381 | 34.5 ± 3.9 | 34.2 ± 4.6 | 0.878 |
